# Light Emitting Diode Therapy Protects against Myocardial Ischemia/Reperfusion Injury through Mitigating Neuroinflammation

**DOI:** 10.1155/2020/9343160

**Published:** 2020-09-03

**Authors:** Songyun Wang, Qinyu Luo, Hui Chen, Jingyu Huang, Xuemeng Li, Lin Wu, Binxun Li, Zhen Wang, Dongdong Zhao, Hong Jiang

**Affiliations:** ^1^Department of Cardiology, Renmin Hospital of Wuhan University, Cardiovascular Research Institute of Wuhan University, Wuhan, Hubei, China; ^2^Department of Cardiology, Shanghai Tenth People's Hospital, Tongji University School of Medicine, Shanghai, China

## Abstract

**Background:**

Neuroinflammation plays a key role in myocardial ischemia-reperfusion (I/R) injury. Previous studies showed that light-emitting diode (LED) therapy might improve M2 microglia activation and brain-derived neurotrophic factor (BDNF) expression, thereby exerting anti-inflammatory effects. Therefore, we hypothesized that LED therapy might reduce myocardial I/R injury by neuroinflammation modulation.

**Objective:**

To explore the effect of LED therapy on myocardial I/R-induced injury and seek the underlying mechanism.

**Methods:**

Thirty rats were randomly divided into three groups: Control group (without LED treatment or myocardial I/R, *n* = 6), I/R group (with myocardial I/R only, *n* = 12), and LED+I/R group (with myocardial I/R and LED therapy, *n* = 12). Electrocardiogram was recorded continuously during the procedure. In addition, brain tissue was extracted for BDNF, Iba1, and CD206 analyses, and heart tissue for myocardial injury (ischemic size and infarct size), IL-4 and IL-10 mRNA analysis.

**Results:**

In comparison with the I/R group, the ischemia size and the infarct size were significantly attenuated by LED therapy in the LED+I/R group. Meanwhile, the microglia activation induced by I/R injury was prominently attenuated by LED treatment either. And it is apparent that there was also an increase in the beneficial neuroinflammation markers (BDNF and CD206) in the paraventricular nucleus (PVN) in the LED+I/R group. Furthermore, the anti-inflammatory cytokines, IL-4 and IL-10, were greatly decreased by I/R while improved by LED treatment in myocardium.

**Conclusion:**

LED therapy might reduce neuroinflammation in PVN and decrease myocardium injury by elevating BDNF and M2 microglia.

## 1. Introduction

Myocardial infarction (MI) is a major cause for the sudden death of patients. Currently, early successful blood restoring is the most effective approach for reducing the myocardial injury of patients with acute myocardial infarction (AMI). However, reperfusion therapy itself can cause damage to myocardial cells and result in the myocardial injury, and no effective therapy for preventing this has been shown [1, 2]. Therefore, exploring a novel method is pressing. Previous studies have shown that neuroinflammation in PVN, the cardiovascular regulatory center in the brain, was involved in many cardiovascular diseases such as myocardial infarction, myocardial I/R, and hypertension [3–7]. Inhibition of neuroinflammation in PVN, however, might improve the outcome of these diseases [8–10].

Recently, previous studies have shown that both M2 microglia and BDNF exert antineuroinflammatory effects [11–14]. Furthermore, LED therapy has been widely used in neurological and brain injury treatment [15, 16]. Moreover, studies have shown that LED therapy might increase M2 microglia and BDNF in the brain, thereby decreasing the ischemic stroke injury [17, 18]. Therefore, we hypothesized that LED therapy might attenuate myocardial I/R induced myocardium injury by neuroinflammation modulation in PVN via elevating BDNF and M2 microglia.

## 2. Methods

### 2.1. Animal Preparation

Thirty Sprague-Dawley rats (250-300 g) were enrolled in the study. They were raised in a temperature-controlled room with abundant supplies of food and water. All experimental procedures were approved by the Animal Ethics Committee of Wuhan University (approval number 2015-0029) and met the standard of the National Institutes of Health Laboratory Animal Care and Use. Rats were anesthetized with an intraperitoneal injection of sodium pentobarbital (40 mg/kg), and body surface electrocardiogram was continuously monitored during the experiment. The pain reflexes (estimated by pinching toes) were observed to ensure the anesthetic depth. The rats were mechanically ventilated with 70 beats/min of respiratory rate.

### 2.2. LED

A tailor-made LED device (610 nm, Convergence Technology, Wuhan, China) was used to deliver light stimulation to the PVN in the present study. The accurate position of PVN was determined by the rat brain atlas of Paxinos and Watson [19] as follows: 2.2 mm posterior and 1.9 mm lateral to bregma, and 7.3 mm below the skull surface. Rats in the LED+I/R group were treated with LED illumination (610 nm, 1.7 mW/cm^2^, 2.0 J/cm2) from 30 minutes before the occlusion of LAD to the end of the experiment, while in the I/R group and the Control group, rats received sham illumination. All these rats received LED or sham LED illumination with their head hair shaved, and no invasive operation on cephalosome was taken during the whole procedure.

### 2.3. Myocardial Ischemia-Reperfusion Model

For the I/R group and the LED-T+I/R group, thoracotomy was made at the fourth intercostal space with the intercostal muscles dissected to expose the heart. Left anterior descending (LAD) coronary artery ligation was made between arterial cone and left atrial appendage with a monofilament 6-0 suture and a piece of polyethylene tube. Reperfusion was done at 30 minutes after LAD ligation by cutting the suture. ST segment or T wave change on electrocardiogram was taken as the verification of ischemia. Rats in the Control group received similar operations but no LAD occlusion.

### 2.4. Immunofluorescence Staining

At the end of the experiment, the PVN tissues of all rats were obtained, sectioned into 5 *μ*m slices, and blocked with 5% bovine serum albumin for 30 minutes at room temperature. They were then incubated overnight with primary antibody Iba-1 (1 : 200, GB12105, Servicebio) or BDNF (1 : 200; Google Bio#GB11240) or CD206 (1 : 200; Google Bio#GB11062) in a wet box at 4°C. Afterwards, sections were rinsed with PBS and incubated with the corresponding Cy3-labelled secondary antibody (1 : 300, Servicebio) in the dark for 50 minutes at room temperature. The nuclei were stained with 4′,6-diamidino-2-phenylindole (DAPI) (G1012, Servicebio). Images were captured with a fluorescence microscope and handled by Image-Pro Plus 6.0 software (Media Cybernetics, Rockville, MD, USA). The positive area of Iba-1-positive, BDNF, and CD206 cells was counted in 3 serial sections at 400× magnification. The average of the 3 serial sections was taken. Same method was used to measure the area of DAPI.

### 2.5. RNA Isolation and Reverse Transcription-Polymerase Chain Reaction

According to the manufacturer's instruction, RNA from left ventricular tissues of each rat was isolated using Trizol Reagent (G3013, Servicebio). The obtained RNA was retrotranscribed into complementary DNA using the PrimeScript RT reagent Kit (#K1622, Fermentas). And then RT-PCR was done in a reaction system with complementary DNA, forward primer, reverse primer, and FastStart Universal SYBR Green Master (04913914001, Roche) [20]. The sequences of IL-4, IL-10, and GAPDH were shown in Table 1.

### 2.6. Measurement of Myocardial Infarct Size

To determine the hypoperfusion area, 1.0% triphenyltetrazolium chloride (TTC) and 2.0% Evans blue solution were used to evaluate the viable area, area at risk (AAR), and infarct area. Evans blue was injected intravenously at the three-hour after reperfusion. After that, the heart was removed after euthanasia, transferred to -80°C refrigerator for 5-6 minutes, and then cut into 5 transverse slices. The slices were dipped in 1% TTC solution (in double-distilled water pH = 7.4) at 37°C for 20 min, rinsed with PBS, and then stored in formalin for 24 hours. Subsequently, the infarct area and AAR were analyzed with Image-Pro Plus 6.0 software.

### 2.7. Statistical Analysis

Mean ± SEM was shown, and *t* test, one-way ANOVA, or two-way repeated-measures ANOVA with a Bonferroni post hoc test analysis were used for continuous variables. However, when the normality test failed or the sample size was small, the Mann-Whitney rank-sum test was used and the median with interquartile range was shown. GraphPad Prism 7.0 software was applied for analyzing all of the data. *P* ≤ 0.05 was considered as statistically significant difference.

## 3. Results

### 3.1. LED Therapy Reduces Myocardial Injury

**Figure 1:**
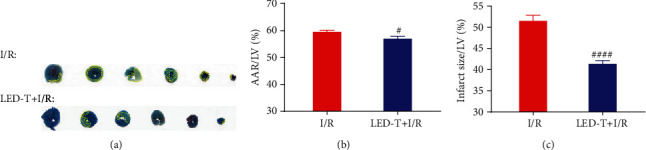
LED therapy reduces myocardial injury. (a) Representative images of Evans blue staining and TTC staining in LED-T+I/R group and I/R group. The blue area represented a normal blood supply area, the red area represented the area at risk (AAR), and the white area represented the infarct area. The infarct area has been outlined with yellow dotted line to make it more obvious. (b, c) Both the AAR/LV (%) and the infarct size/LV (%) in the I/R+LED group were significantly reduced as compared to those in the I/R group. ^#^*P* < 0.05 vs. I/R group. ^####^*P* < 0.0001 vs. I/R group.

Figure 1(a) showed the representative myocardial staining pictures in the I/R and the LED+I/R groups. Figures 1(b) and 1(c) showed that both the AAR/LV (%) and the infarct size/LV (%) in the I/R+LED group were significantly reduced as compared to those in the I/R group (AAR/LV: 57.08 ± 0.90% vs. 59.58 ± 0.62%, *P* < 0.05; infarct size/LV: 41.33 ± 0.82% vs. 51.50 ± 1.34%, *P* < 0.0001).

### 3.2. LED Treatment Improves the Beneficial Function of Microglia

**Figure 2:**
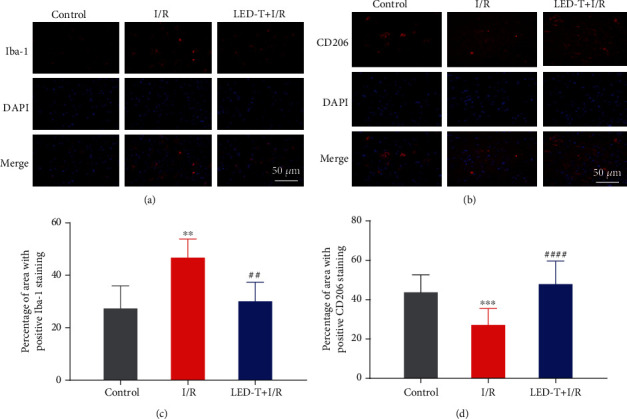
LED treatment improves the beneficial function of microglia. (a, b) Representative immunofluorescence staining for Iba-1 and CD206 in the PVN of rats' brain from all three groups. Scale bar = 50 *μ*m. (c, d) Quantitative analysis of the positive percentage of the area of Iba-1 and CD206 (% of the area of DAPI). ^∗∗^*P* < 0.01 vs. control group. ^∗∗∗^*P* < 0.001 vs. control group. ^##^*P* < 0.01 vs. I/R group. ^####^*P* < 0.0001.

Figures 2(a) and 2(b) showed the representative immunofluorescence staining for Iba-1 and CD206. Iba1, a biomarker for showing the total number of activated microglia, was notably increased in the I/R group as compared to that in the Control group (46.72 ± 3.18% vs. 27.37 ± 3.847%, *P* < 0.01). However, the activation of microglia induced by myocardial I/R injury was significantly decreased by LED therapy (30.1 ± 3.252% vs. 46.72 ± 3.18%, *P* < 0.01) (Figure 2(c)). CD206, which was known as a biomarker for the anti-inflammatory M2 type microglia, was remarkably decreased in the I/R group as compared that into the Control group (27.13 ± 2.51% vs. 43.74 ± 3.15%, *P* < 0.001), whereas it kept a comparable level in the LED+I/R group (47.87 ± 3.04% vs. 27.13 ± 2.51%, *P* < 0.01) (Figure 2(d)).

### 3.3. LED Therapy Improves the Expression of BDNF

**Figure 3:**
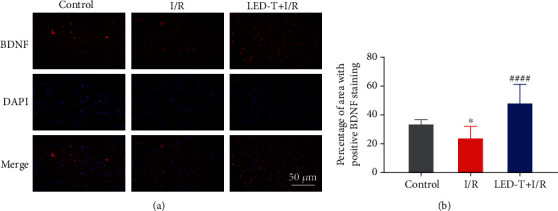
LED therapy improves the expression of BDNF. (a) Representative immunofluorescence staining for BDNF in the PVN of rats' brain from all three groups. Scale bar = 50 *μ*m. (b) Quantitative analysis of the positive percentage of the area of BDNF (% of the area of DAPI). NS, ^∗^*P* < 0.05 vs. control group; ^####^*P* < 0.0001 vs. I/R group.

Figure 3(a) showed the expression of BDNF, which was a neuroprotective biomarker, in the Control, I/R, and LED+I/R groups. As compared to the Control group, the expression of BDNF in the I/R group was decreased from 33.39 ± 3.416% to 23.67 ± 2.107%, while an apparent increase was presented in the LED+I/R group (LED+I/R group vs. I/R group: 47.96 ± 3.845% vs. 23.67 ± 2.107%, *P* < 0.0001) (Figure 3(b)).

### 3.4. LED Therapy Increases the Level of IL-4 and IL-10 in Myocardial Tissue

**Figure 4:**
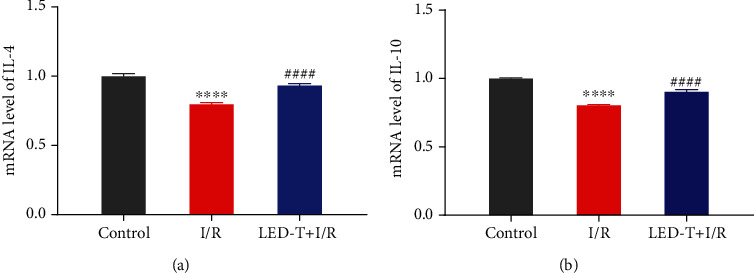
LED therapy increases the level of IL-4 and IL-10 in myocardial tissue (Fold changes relative to the control group). Data represent the mRNA level of target interleukin amount relative to control which is considered 1. (a) The level of IL-4 in the myocardium was significantly reduced by I/R, and this trend was remarkably attenuated by LED therapy. (b) The level of IL-10 in the myocardium was significantly decreased in I/R group as compared to the control group, whereas notably increased with the treatment of LED therapy. ^∗∗∗∗^*P* < 0.0001 vs. the control group, ^####^*P* < 0.0001 vs. I/R group.

Figures 4(a) and 4(b) showed that the level of anti-inflammatory cytokines, IL-4 and IL-10, were prominently reduced by I/R, and this trend was remarkably attenuated by LED therapy either (IL-4: Control group vs. I/R group 1 ± 0.0187 vs. 0.7965 ± 0.0126, *P* < 0.0001; I/R group vs. LED+I/R group: 0.7965 ± 0.0126 vs. 0.9327 ± 0.0145, *P* < 0.0001) (IL-10: Control group vs. I/R group 0.9999 ± 0.0061 vs. 0.8038 ± 0.0057, *P* < 0.0001; I/R group vs. LED+I/R group: 0.8038 ± 0.0057 vs. 0.9018 ± 0.0166, *P* < 0.0001).

## 4. Discussion

### 4.1. Major Findings

Our results showed that both the ischemia size and the infarct size were greatly attenuated in the LED+I/R group as compared to those in the I/R group. Meanwhile, the I/R-induced microglia activation was notably inhibited. In addition, both beneficial neuroinflammation markers, BDNF and CD206, showed a remarkable reduction in the PVN by the therapy either. Moreover, the anti-inflammatory cytokines, IL-4 and IL-10, were significantly decreased by I/R while improved by the LED treatment. All these findings indicated that LED therapy might play a significant role in reducing myocardial I/R injury and mitigating neuroinflammation via elevating M2 microglia and BDNF in PVN.

### 4.2. Myocardial I/R Injury and Neuroinflammation

PVN is the sympathetic neural modulation center of cardiovascular function [21]. A host of studies demonstrated that many cardiovascular diseases, such as myocardial infarction, heart failure, myocardial I/R, and hypertension, were related to neuroinflammation in PVN, and inhibition of such neuroinflammation could improve the outcome of these diseases [9, 22–25]. For example, previous studies suggested that there was an increase of proinflammatory cytokines and activated microglia in the PVN post-MI [3, 8, 26, 27], and the inhibition of activated microglia in PVN by minocycline resulted in smaller infarct size following MI [8]. Consistently, our recent studies also demonstrated a significant increase of activated microglia in AMI and myocardial I/R rat model and showed that the inhibition of activated microglia might lead to a reduction of inducibility of ventricular arrhythmias(VAs) caused by AMI and myocardial I/R. However, the underlying mechanism of neuroinflammation is unclear.

Microglia, resident immune cells of the CNS, are the front-line defense against any central nervous system(CNS) injuries and the key player in both acute and chronic neuroinflammation [28, 29], they will turn to “activated” states when insulted with any kinds of damage. The “activated” microglia comprise two subtypes, the M1 microglia and the M2 microglia. M1 microglia are typified by the production of proinflammatory cytokines and reactive oxygen species while M2 microglia, which release abundant anti-inflammatory factors and neurotrophin, are contrarily specialize in anti-inflammation and neural protection [30]. The activation of microglia is initially protective to the CNS; it is believed to be the fundamental response to CNS injuries. However, excessive mobilization of M1 microglia leading to uncontrolled neuroinflammation turns out to be a big threat in many neuroinflammation-related diseases and the anti-inflammatory effect generated by M2 microglia is usually unsufficient to halt this trend [28, 29]. Therefore, substantial studies have suggested that the proliferation of M2 microglia had powerful potential in mitigating the neuroinflammation and would be a promising target for many neuroinflammation-related diseases. Some studies have confirmed this speculation in several neuroinflammation-related diseases models, such as stroke, traumatic brain injury, and Alzheimer's disease [31–33]. BDNF is the most abundant neurotrophic factor in the brain, which specializes in synapses formation, maintenance of neural plasticity, and neurite differentiation and proliferation [34]. Recent studies found that it also functioned as an anti-inflammatory factor in CNS [35, 36]. Ji et al. demonstrated that the application of BDNF in CNS could result in the promotion of M2 microglia/macrophage and the reduction of several proinflammatory cytokines, such as IL-1*β* and TNF-*α* [37]. Considering that M2 microglia is one of the major sources of BDNF [38], it seems that the elevation of BDNF and M2 microglia could mutually reinforce to create a positive feedback for antineuroinflammation. All these findings have well indicated that improving the neuroinflammation protection might be a promising therapeutic method for myocardial I/R injury and that M2 microglia and BDNF might be ideal targets for such protection.

### 4.3. LED Therapy for I/R Neuroinflammation Mitigation and Myocardial Injury Reduction

Photobiomodulation (PBM) therapy was beneficial for reducing pain, anti-inflammation, and tissue repair [39]. Compared with the traditional therapeutic method, PBM therapy shows unparalleled advantages in noninvasiveness, which gives a broad prospect in the promotion of clinical application, such as traumatic brain injury, stroke, and depression [40, 41]. Recent studies have demonstrated that PBM could exert a prominent effect on inhibiting neuroinflammation via reducing proinflammatory factors and promoting M2 microglia polarization [42, 43] and that it could increase neurogenesis via elevating neurotrophic factors, such as BDNF [44]. Meanwhile, recent studies have demonstrated that the elevation of BDNF could also exert an anti-inflammatory effect as we have mentioned above. In the present study, we have observed a notable increase of M2 microglia and BDNF, which demonstrates that LED illumination leads to M2 polarization and further strengthens the tone of anti-inflammation by elevating BDNF. All these implicate that LED illumination may greatly improve the protective M2 microglia and BDNF, thereby mitigating neuroinflammation in PVN.

Moreover, we have found a considerable reduction of myocardial infarction size and a significant elevation of anti-inflammatory cytokines in the myocardium in the LED+I/R group compared with those in the I/R group. Previous studies have shown that the inhibition of neuroinflammation could depress sympathetic activity [45, 46] which serves not only the consequences of many cardiovascular diseases but also the primary mechanism to their deterioration. Furthermore, many studies have shown that the weakening of central sympathetic tone could lead to the improvement of peripheral inflammatory states in cardiovascular diseases, which could ameliorate cardiac dysfunction, myocardial infarction, and cardiac remodeling [47–49]. All these demonstrate that the underlying mechanism of LED therapy on myocardial I/R injury reduction may owe to the interaction among neuroinflammation, sympathetic activity, and peripheral inflammation.

## 5. Conclusion

In the present study, we demonstrated that LED therapy could effectively modulate neuroinflammation in PVN via elevating M2 microglia and BDNF, thereby reducing myocardial I/R injury. The modulation of neuroinflammation is a viable and promising therapeutic method for myocardial I/R injury, and M2 microglia and BDNF are promising targets for such modulation.

## Figures and Tables

**Table 1:** Primer information of genes validated by TaqMan RT-PCR.

Gene name	Accession no.	Primer sequence	Amplicon size (bp)
IL-4	NM_201270.1	S: 5′-CTGTCACCCTGTTCTGCTTTCTC-3′	105
		A: 5′-TTTCTGTGACCTGGTTCAAAGTGT-3′	
IL-10	NM_012854.2	S: 5′-CACTGCTATGTTGCCTGCTCTT-3′	100
		A: 5′-GTCTGGCTGACTGGGAAGTGG-3′	
GAPDH	NM_017008.4	S: 5′-CTGGAGAAACCTGCCAAGTATG-3′	138
		A: 5′-GGTGGAAGAATGGGAGTTGCT-3′	

## Data Availability

The data used to support the findings of this study are available from the corresponding author upon request.

## Abbreviations

LED: Light-emitting diode
I/R: Ischemia/reperfusion
BDNF: Brain-derived neurotrophic factor
PVN: Paraventricular nucleus
MI: Myocardial infarction
AMI: Acute myocardial infarction
VAs: Ventricular arrhythmias
CNS: Central nerves system
PBM: Photobiomodulation.
